# Improving Confidence in Identifying and Managing Perinatal Mental Health Risks Amongst Acute Clinical Services (First Response Services (FRS), Intensive Home Treatment Team (IHTT) and Acute Liaison Psychiatric Services (ALPS) Within the Bradford District Care NHS Foundation Trust

**DOI:** 10.1192/bjo.2025.10328

**Published:** 2025-06-20

**Authors:** Oluwatobi Adejimi, Lian Chua, Lisa Massheder, Lisa Milne

**Affiliations:** Bradford District Care NHS Foundation Trust, Bradford, United Kingdom

## Abstract

**Aims:** The MBBRACE (confidential enquiry into maternal deaths in the UK) report tells us women in the perinatal period are a high-risk group and that this is not always recognized. The unique nature and complexities of perinatal illness that can present in these vulnerable groups requires thinking about both mother and baby. MBBRACE report tells us that we must recognize these risks and there must be a lowered threshold for accessing and referring to Mother and Baby Units and how to do this out-of-hours. As a gateway to mental health services, FRS, IHTT, ALPS services must have perinatal awareness and risk training as mandatory.

This quality-improvement project aims to improve clinical skills, service delivery and patient safety

**Methods:** We aimed to first identify the knowledge level of the FRS, IHTT and ALPS teams, if they had attended any perinatal training before (in the last 12 months, longer than 12 months or never) and asking their areas of developmental needs. This was through interactive online questionnaire tools (pre-intervention questionnaires). Then teaching sessions on perinatal mental health risks delivered to these teams. A post-intervention questionnaire to objectively measure improvements in skill and confidence.

**Results:** Pre-questionnaire revealed for majority of responders, the last time they attended a perinatal mental health teaching was 12 months ago – 3 years (barring ALPS, who had a teaching few months ago). Confidence level pre-intervention (teaching) – 14% (not confident), 28% (slightly confident), 51% (somewhat confident) and 7% (confident). Post-intervention – 33%(confident), 50% (very confident), 17% (extremely confident).

The outcome was that confidence levels in identifying and managing perinatal mental health risks were significantly increased.

**Conclusion:** Having regular inter-team teaching sessions on perinatal mental health risks (including perinatal red flags) increases the confidence levels of first line/Acute Clinical Services in identifying and promptly managing these perinatal mental health risks. This would in turn reduce perinatal mental health safety incidences or near misses.

